# Impact of polyunsaturated fatty acids on patient-important outcomes in children and adolescents with autism spectrum disorder: a systematic review

**DOI:** 10.1186/s12955-020-01284-5

**Published:** 2020-02-17

**Authors:** Franco De Crescenzo, Gian Loreto D’Alò, Gian Paolo Morgano, Silvia Minozzi, Zuzana Mitrova, Rosella Saulle, Fabio Cruciani, Francesca Fulceri, Marina Davoli, Maria Luisa Scattoni, Francesco Nardocci, Holger Jens Schünemann, Laura Amato, on behalf of the ISACA guideline working group, Francesco Nardocci

**Affiliations:** 1 Department of Epidemiology, Lazio Regional Health Service, Via Cristoforo Colombo, 112, 00154 Rome, Italy; 2 Department of Psychiatry, University of Oxford, Oxford, UK; 3 Pediatric University Hospital-Department (DPUO), Bambino Gesù Children’s Hospital, Rome, Italy; 4 School of Hygiene and Preventive Medicine, University of Rome Tor Vergata, Rome, Italy; 5 Department of Health Research Methods, Evidence and Impact (formerly Clinical Epidemiology and Biostatistics), McMaster GRADE Centre, McMaster University, Hamilton, Canada; 6 Research Coordination and Support Service, Istituto Superiore di Sanità, Viale Regina Elena 299, 00161 Rome, Italy; 7 Michael G DeGroote Cochrane Canada Centre, McMaster University, Hamilton, Canada; 8 Department of Medicine, McMaster University, Hamilton, Canada

**Keywords:** Autism spectrum disorder, Polyunsaturated fatty acids, Systematic review, Meta-analysis, Children, Adolescents

## Abstract

**Background:**

Recent randomized controlled trials (RCTs) claimed PUFAs to be effective for autism spectrum disorder (ASD) but international guidelines have not considered yet this body of evidence. Our aim was to assess the effectiveness of PUFAs in children and adolescents with ASD, for the Italian national guidelines on the management of ASD in children and adolescents.

**Methods:**

We performed a systematic review and meta-analysis of RCTs comparing PUFAs versus placebo or a healthy diet for the treatment of ASD in children and adolescents. The outcomes considered were deemed by the guideline panel to be highly relevant to children and adolescents with ASD and to their caregivers. The outcomes included hyperactivity, quality of sleep, self-harm, aggression, irritability, anxiety, attention, adaptive functioning, social interaction, restricted and repetitive interests and behavior, communication, hyperactivity and disruptive behaviors coexistent with core symptoms. The risk of bias of the included studies was assessed with the Cochrane tool, and the rating of the confidence in the effect estimates according to the Grading of Recommendations Assessment, Development and Evaluation (GRADE) approach.

**Results:**

We included 9 studies with 405 participants. The strength of evidence ranged from low to very low. Six studies included preschoolers and school-age children, three studies included both children and adolescents. The majority of participants were males (83.8%), with a mean age of 6.7 years. PUFAs were superior compared to placebo in reducing anxiety in individuals with ASD (SMD -1.01, 95% CI − 1.86 to − 0.17; very low certainty of evidence). Moreover, PUFAs worsened quality of sleep compared to a healthy diet (SMD 1.11, 95% CI 0.21 to 2.00; very low certainty of evidence). PUFAs were not better than placebo in reducing aggression, hyperactivity, adaptive functioning, irritability, restricted and repetitive interests and behaviors and communication. Effects on some critical outcomes such as sleep, self-harm and disruptive behavior are currently unknown. The main limitations were the small number of participants included in the RCTs and the dosage which varied greatly (from 200 mg/day to 1540 mg/day), making it difficult to address causal inference.

**Conclusions:**

PUFAs did not show evidence of effect in children and adolescents with ASD and the certainty of evidence as measured with the GRADE was low to very low. Further research is needed on this topic because the available evidence is inconclusive.

## Introduction

Autism spectrum disorder (ASD) is characterized by abnormal neurodevelopment, with core symptoms consisting in persistent alterations in social interaction and communication, and restricted and repetitive interests and behaviors that cause reduced functioning, regardless of intellectual ability [1].

The prevalence of ASD in Italy is about 1.14–1.3% [2, 3]), and its prevalence in the world is between 1 and 2% [4]. A recent Italian study found a male: female ratio of about 4:1 [5], with 48% of children being affected by intellectual disability [5], data are consistent with the international literature [6].

Poly-unsaturated fatty acids (PUFAs) contain at least two carbon-carbon double bonds in their carboxylic chain, and can be classified according to the distance of the first double bond from the methyl group placed at the end of the molecule, into omega-3, omega-6 and omega-9 (the latter is not essential in humans because they can be synthesized from carbohydrates or other fatty acids). Fish oils are rich in omega-3, plants are rich in omega-6, and two PUFAs, α-linolenic acid (an omega-3 fatty acid) and linoleic acid (an omega-6 fatty acid) are essential nutrients in humans [7].

Eicosapentaenoic acid (EPA) and docosahexaenoic acid (DHA), are omega-3 fatty acids thought to favor a reduction in the synthesis of pro-inflammatory mediators. This effect has supported their use in the secondary prevention of hypertension, coronary artery disease, type 2 diabetes and in some other diseases [8], although their effect is controversial [7, 9, 10]. The role of EPA and DHA in disorders of the central nervous system has been extensively investigated in the last two decades [8]. The rationale behind the use of these agents in psychiatric disorders would be their primary action in producing modifications of the synaptic membrane, with implications in the transmission and transduction of the signal [8, 11]. Magnetic resonance imaging studies suggested that a reduced functional connection of long-distance brain areas is related to difficulties in social interactions in children and adolescents with autism spectrum disorder [12]. In mental health, EPA and DHA have been studied for the therapy of attention deficit hyperactivity disorder (ADHD), ASD, unipolar and bipolar affective disorders, anxiety disorder, obsessive-compulsive disorder, aggression, hostility, impulsivity, borderline personality disorder, substance use and anorexia nervosa [8, 13].

The risk of serious adverse events such as stroke, pulmonary embolism, and bleeding following PUFAs administration is still unclear, notwithstanding some recent RCTs showed a small increase of these events in the PUFAs arms [7, 14]. When fish oil is ingested for a long period of time (several months), it is better to ingest vitamin E (antioxidant) together, in preparation for lipid peroxidation. Also, as the toxicity of vitamin A or D could be increased, FDA recommends not ingesting more than 3 g of fish oil-derived omega-3 fatty acids per day [15]..

The goal of this systematic review was to assess the efficacy and safety of PUFAs in children and adolescents with ASD.

## Methods

This systematic review was performed to support the development of the Italian National Institute of Health (ISS) guidelines for the diagnosis and management of children and adolescents with ASD. The ISS guideline group for the diagnosis and management of autism spectrum disorder, comprised of a multidisciplinary panel including caregivers of children/adolescents with ASD, formulated 15 questions for developing evidence-based health recommendations [16, 17] in accordance with the recently published ISS methodological manual for clinical practice guidelines (GL) development [18]. The Evidence Review Team together with the ISS principal investigator and the GL chairs decided to include two more questions for training the panel members on the pathway leading to the recommendations.

Using the GRADE approach, the panel began its work agreeing on a recommendation addressing the impact of PUFAs on patient-important outcomes in children and adolescents with autism spectrum disorder; a common question for this population.

### The questions

Should PUFAs versus placebo be used for the treatment of children and adolescents with ASD?

Should PUFAs versus healthy diet be used for the treatment of children and adolescents with ASD?

### Population

Children and adolescents aged 0–18 years, of both genders, with a primary diagnosis of autism spectrum disorder. A concurrent secondary diagnosis of another health disturbance was not considered as an exclusion criterion.

### Intervention

Any type and any dose of PUFAs, including eicosapentaenoic acid, docosahexaenoic acid, and α-linolenic acid. We will include also studies in which fatty acids will be used as adjunctive treatment (for example, indicated in addition to behavioral or pharmacological interventions).

### Comparisons

- Placebo or no intervention.
- Healthy diet.

### Outcomes

The outcomes considered in this meta-analysis were deemed by the guideline panel to be highly relevant to children and adolescents with ASD. They were identified in accordance with the methods described in the ISS manual [18] and are the result of a group process conducted using the guideline development tool GRADEpro [19], which includes outcomes’ generation and rating on a 9-point scale. Outcomes with a mean rating score from 6.33 to 9 were considered critical, from 3.33 to 6.32 important, from 1 to 3.32 not important for decision-making.

To measure the efficacy of the treatment, we assessed the following outcomes:

- Hyperactivity (critical),
- Quality of sleep (critical),
- Self-harm (critical),
- Aggression (critical),
- Irritability (critical),
- Anxiety (critical),
- Attention (critical),
- Adaptive functioning (critical),
- Social interaction (important),
- Restricted and repetitive interests and behavior (important),
- Communication (important),
- Hyperactivity and disruptive behaviors coexistent with core symptoms (important).

In addition, the evidence review team measured the tolerability of the treatment through the following outcomes:

- Discontinuation due to any cause (not important),
- Number of adverse events (not important).

### Types of studies included

Randomized controlled trials comparing PUFAs with placebo or any other intervention in the treatment of autism spectrum disorder were included. Quasi-randomized trials, such as those allocating by using alternate days of the week, and open-label trials were excluded. For trials that had a crossover design only results from the first randomization period were considered.

### Literature search

A comprehensive computer literature search of the CENTRAL, PubMed/Medline, Embase, PsycINFO, Web Of Science databases was carried out up to October 2018. We also searched for ongoing clinical trials and unpublished trials. The full search strategy used is available in the supplementary materials, Additional file 1. No date limit and no language restrictions were used.

### Study selection and data extraction

Two reviewers (FDC, GD) independently screened titles and abstracts of all publications that were obtained by the search strategy. The same authors independently assessed the full text of potentially-relevant studies for inclusion. Disagreement was resolved by a consensus meeting or by a third reviewer (LA).

Two reviewers (FDC, GD) independently extracted data. We used a structured data abstraction form to ensure consistency of appraisal for each study. Information extracted included study characteristics (such as lead author, publication year, journal), participant characteristics (age range, setting, diagnosis), intervention details (such as dose ranges, mean doses of study drugs), length of follow up and outcome measures of interest.

### Data analysis

Data were entered and analyzed using RevMan 5.3 software. Continuous outcomes were analyzed using standardized mean difference (SMD) with 95% confidence intervals because different scales were used in the included studies. We combined data using the random effect model because a certain degree of heterogeneity was expected among trials [20]. In interpreting SMD values, we considered SMD “small” if < 0.40, “moderate” from 0.40 to 0.70, and “large” if > 0.7. We analyzed dichotomous outcomes by calculating the risk ratio (RR) for each trial with the uncertainty in each result being expressed with 95% confidence interval (CI). Heterogeneity between studies has been investigated by the Q-test, by I-squared statistic (I-squared equal to or more than 50% was considered indicative of heterogeneity), and by visual inspection of the forest plots.

### Risk of bias and overall certainty of evidence assessment

Two authors independently (FDC, GD) assessed the risk of bias in the included studies using the tool described in the Cochrane Handbook for systematic reviews of interventions as a reference guide [21]. The following domains were assessed:

1. sequence generation;
2. allocation concealment;
3. blinding;
4. incomplete outcome data;
5. selective reporting;
6. other bias (e.g. funding source, baseline imbalance, interventions insufficiently well delivered).

A ‘Risk of bias’ table was created for the included studies, which indicates the study’s performance in each of the above domains. For each domain, a judgment was assigned in terms of low risk of bias; high risk of bias; unclear risk of bias.

The main results of the review were presented in ‘Summary of findings’ (SoF) tables, as recommended by Cochrane [22]. We produced the SoF tables for estimates based on the methodology developed from the Grading of Recommendations Assessment, Development and Evaluation (GRADE) Working Group [23]. For more details, see [24, 25]. We rated the confidence in the effect estimates considering study limitations, indirectness, inconsistency, imprecision of effect estimates, and risk of publication bias. According to the software GRADEpro GDT 2014, four levels of certainty in the evidence were assigned: high, moderate, low, very low.

Three authors (FDC, SV, RS) applied the GRADE approach to evaluating the certainty of evidence for the outcomes considered as “critical”, “important”, or “not important” from the members of the panel:

- Efficacy (hyperactivity, quality of sleep, self-harm, aggression, irritability, anxiety, attention, adaptive functioning, social interaction, restricted and repetitive interests and behavior, communication, hyperactivity and disruptive behaviors coexistent with core symptoms);

- Tolerability (discontinuation due to any cause, number of adverse events).

## Results

### Selected studies

**Fig. 1:**
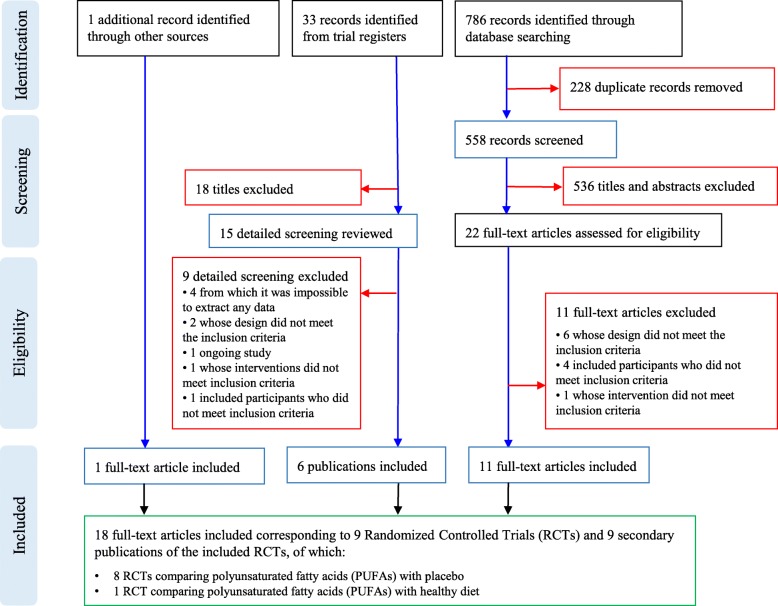
Flow chart

From databases searches, we retrieved 786 citations of which 228 were removed, being duplicates. Of the 558 remaining documents, 22 studies were evaluated in full text as potentially relevant. Of these, eleven were excluded. Among the excluded, six studies were not RCTs [26–31], four studies included preterm infants between 18 and 36 months with a high risk of ASD [32–35], one study assessed an intervention that did not meet inclusion criteria [36]. We retrieved further 33 records from trial registers, 15 of which were evaluated in full text. We found four completed clinical trials of which we were unable to obtain any result [37–40]; two trials whose design did not meet inclusion criteria [41, 42]; one ongoing study [43]; one trial whose intervention did not meet inclusion criteria [44]; and one trial whose participants did not meet inclusion criteria [45]. We also retrieved one full-text document from other sources [46] (Fig. 1).

Finally, a total of 9 studies with 405 participants (18 documents), were included (see Additional file 7: references for included and excluded trials).

### Study characteristics

Six studies (66.7%) included preschoolers and school-age children, while three studies (33.3%) included both children and adolescents. The majority of participants were males (83.8%), with a mean age of 6.7 years. In seven studies the diagnosis was performed using the DSM-IV criteria, while one study used the DSM-5 criteria [46] and another study based the diagnosis on parent reports [47]. Seven studies reported the use of scales for support in the diagnosis, such as the Autism Diagnostic Interview-Revised (ADI-R), Autism Diagnostic Observation Scale (ADOS), Childhood Autism Rating Scale (CARS), Social Communication Questionnaire (SCQ). All individuals included in the selected studies were outpatients.

Eight studies compared PUFAs vs placebo [12, 46–52], and one vs healthy diet [53].

We considered healthy diet as something closed to no intervention because is part of standard clinical care.

Overall, 201 participants were randomly assigned to PUFAs, 161 to placebo, 13 to a healthy diet, and 30 to other interventions (i.e. Vitamin D supplementation). The mean study sample size was 45 participants, ranging between 13 [48] and 110 [46] participants. Two studies recruited patients from Europe, five from North America, one from Asia and one from Oceania. Study median duration was twelve weeks (range: 6–52).

The scales used to measure the outcomes of interest included the Aberrant Behavior Checklist (ABC), the Behavior Assessment System for Children (BASC), the Clinical Global Impression-Improvement scale (CGI-I), the Clinical Global Impression-Severity scale (CGI-S), the Expressive Vocabulary Test (EVT), the Mullen Scales of Early Learning, the Preschool Language Scale (PLS), the Peabody Picture Vocabulary Test (PPVT), the Social Responsiveness Scale (SRS), the Vineland Adaptive Behavior Scale (VABS).

Regarding “hyperactivity” outcome, data were extracted from ABC – hyperactivity subscale [9, 42–45]; for the outcome “quality of sleep”, we used the CBCL – sleep subscale [51]; for “aggression”, we selected BASC – externalizing subscale [49] and CBCL – aggression subscale [53]; for the outcome “irritability”, we selected ABC – irritability subscale [9, 12, 46–48]; for the outcome “anxiety”, we extracted data regarding the BASC – internalizing subscale [49] and CBCL – anxious/dependent subscale [53]; for the outcome “attention”, we selected CBCL – attention subscale [53]; for “adaptive functioning”, we used the BASC – adaptive skill subscale [49] and the item social skills - parents assessed of the subscale adaptive skills of the BASC [52]; for the outcome “social interaction”, we selected the SRS total score [12, 47, 49, 51], and, when this scale was not assessed, the ABC - social withdrawal subscale [46, 48], or the BASC – withdrawn subscale [53]; for the outcome “restricted and repetitive interests and behaviors” we selected the ABC – stereotypy subscale [46–49], and from SRS - autistic mannerisms subscale [51]; for “communication” outcome, we selected data from ABC – inappropriate speech subscale [46–48], from EVT [49], from SRS - social communication subscale [12, 51], and from MSEL – expressive language subscale [53].

As regards to the composition of PUFAs, in five trials a combination of EPA and DHA were administered, while in four studies only DHA was prescribed. Doses of EPA ranged from 693 mg [51] to 840 mg/day [48], while doses of DHA ranged from 200 mg [52] to 722 mg/day [46].

Overall PUFAs dosage varied greatly in the studies analyzed, with a median dose of 1155 mg/day, ranging from a minimum of 200 mg/day [52] to a maximum of 1540 mg/day [48]. Dropout rates varied between 7.7% [48] and 29.6% [49]; in two studies [12, 53] dropouts were not clearly reported. We reported full clinical and demographic characteristics and description of interventions in Table 1.

**Table 1:** Characteristics of included Randomized Controlled Trials

Study, year	Country	Diagnosis	Diagnostic criteria	Intervention (n)	Control (n)	Duration of intervention (weeks)	Age mean (SD) (years)	Female (%)	Outcomes	Funding source
Amminger, 2007 [44]	Austria	Autistic disorder	DSM-IV; ADI-R; ADOS	EPA 0.84 g/day, DHA 0.7 g/day, and vitamin E (7 mg/day) (7)	Placebo (6)	6	10.4 (3.2); range: 5–17 years	0 (0%)	ABC, adverse events	Omega Protein Cooperation, Houston, Texas
Bent, 2011 [45]	USA	Autistic spectrum disorder	DSM-IV-TR; ADOS; SCQ	EPA 0.7 g/day and DHA 0.46 g/day (14)	Placebo (13)	12	5.8 (1.7); range: 3–8 years	3 (11.1%)	ABC, Communication (PPVT, EVT), Social interaction (SRS); Behaviours (BASC); Global changes (CGI-I)	Autism Speaks; the Higgins Family Foundation; The Emch Foundation; The Taube Foundation; NIH/NCRR UCSF-CTSI UL1RR024131; MIND Institute
Bent, 2014 [43]	USA	Autistic spectrum disorder	Parent-report; Social Communication Questionnaire (SCQ)	EPA 0.7 g/day and DHA 0.46 g/day (29)	Placebo (28)	6	7.2 (1.1); range: 5–8 years	7 (12.3%)	ABC, parent and teacher ratings, Social interaction (SRS), Global changes (CGI-I)	Simons Foundation (SFARI 206484)
Johnson, 2010 [49]	USA	Autistic spectrum disorder; Pervasive Developmental Disorder, NOS	DSM-IV; ADOS	DHA 0.4 g/day (10)	Healthy diet (no treatment) (13)	12	3.4 (0.7); range: NR	NR	Child Behavior Checklist, MSEL AGS edition, direct behaviour observation measure, adverse events	John F. & Nancy A. Emmerling Fund / The Pittsburgh Foundation
Mankad, 2015 [46]	Canada	Autistic spectrum disorder	DSM-IV; ADOS; Autism Diagnostic Interview - Revised (ADI-R)	EPA and DHA (0.75 to 1.5 g/day) (18)	Placebo (19)	24	3.7 (1.5); range: 2–5 years	10 (26.3%)	Behaviors (BASC-2, PDDBI); Adaptive skills (VABS-II); language (PLS-4); global improvement (CGI-I); adverse events	Alva Foundation
Mazahery, 2018 [42]	New Zealand	Autistic spectrum disorder	DSM-V	DHA (0.722 g/day) (28) Vitamin D (2000 IU/day) (30) DHA (0.722 g/day) plus Vitamin D (2000 IU/day) (25)	Placebo (28)	52	5.3 (1.4); range: 2.5–8 years	13 (17.8%)	ABC	Massey University Strategic Innovation Fund, Massey University, New Zealand; Douglas Nutrition, Pty. Ltd
Parellada, 2017 [47]	Spain	Autistic spectrum disorder	DSM-IV-TR (Pervasive Developmental Disorder)	EPA (0.578 to 0.693 g/day), DHA (0.385 to 0.462 g/day) and vitamin E (1.6 to 2.10 mg/day) (40)	Placebo (37)	8 (crossover)	9.7 (3.6); range: 5–17 years	11 (16.2%)	Communication (SRS); Global severity (CGI-S); Proportion of ω3 PUFAs in erythrocyte (RBC) membranes [the ratio of AA to DHA and EPA (AA/DHA, AA/EPA) and ω3/ω6]; Plasma total antioxidant oxidative status (TAS);	Spanish Ministry of Economy and Competitiveness, Instituto de Salud Carlos III (FIS EC07/90076); (CB/07/09/0023), and the Alicia Koplowitz Foundation(FAK 2017)
Voigt, 2014 [48]	USA	Autistic spectrum disorder	DSM-IV; CARS	DHA (0.2 g/day) (24)	Placebo (24)	26	6.1 (2.0); range: 3–10 years	8 (16.7%)	ABC; Global improvement (CGI-I); behaviour (BASC), depression (CDI)	Mayo Foundation; Martek Biosciences Corporation (Columbia, MD)
Yui, 2012 [9]	Japan	Autistic disorder or Asperger disorder	DSM-IV; ADI-R	DHA (0.24 g/day), arachidonic acid (0.24 g/day), astaxanthin (0.96 mg/day) (7)	Placebo (6)	16	14.6 (6.0); range: 6–28 years	1 (7.7%)	ABC; Social interaction (Social Responsiveness Scale)	Ministry of Education, Culture, Sports, Science and Technology, Japan; Sawa Hospital, Osaka, Japan.

**Legend**: *ABC* Aberrant Behavior Checklist, *ADI-R* Autism Diagnostic Interview—Revised, *ADOS* Autism Diagnostic Observation Scale, *BASC* Behavioral Assessment System for Children, *CARS* Childhood Autism Rating Scale, *CDI* Children’s Depression Inventory, *CGI-I* Clinical Global Impression-Improvement scale, *CGI-S* Clinical Global Impression-Severity scale, *DHA* docosahexaenoic acid, *EPA* eicosapentaenoic acid, *EVT* Expressive Vocabulary Test, *MSEL* Mullen Scales of Early Learning, *NR* Not reported, *PDDBI* Pervasive Developmental Disorder- Behavioral Inventory, *PLS-4* Preschool Language Scale, Fourth Edition, *PPVT* Peabody Picture Vocabulary Test, *SCQ* Social Communication Questionnaire, *SRS* Social Responsiveness Scale, *VABS-II* Vineland Adaptive Behavior Scales, Second Edition

### Data analysis and overall certainty of evidence assessment

We presented the forest plots for specific outcomes for the comparisons PUFAs versus placebo in Additional file 3 and PUFAs versus healthy diet in Additional file 4. Summary of findings for the comparisons PUFAs versus placebo and PUFAs versus healthy diet are presented in Table 2 and Table 3. We also presented the evidence profiles for the comparisons PUFAs versus placebo and PUFAs versus healthy diet in Additional file 5 and Additional file 6, respectively.

**Table 2:** Summary of Findings (SoF) for the comparison PUFAs versus placebo

Summary of findings:						
Should polyunsaturated fatty acids versus placebo be used for the treatment of children and adolescents with autism spectrum disorder?						
Patient or population: children and adolescents with autism spectrum disorder						
Setting: outpatients						
Intervention: polyunsaturated fatty acids						
Comparison: placebo						
Outcomes	Anticipated absolute effects^*^ (95% CI)		Relative effect (95% CI)	№ of participants (studies)	Certainty of the evidence (GRADE)	Comments
	Risk with placebo	Risk with polyunsaturated fatty acids				
Hyperactivity	–	SMD **0.27 lower** (0.6 lower to 0.06 higher)	–	146 (5 RCTs)	⨁⨁◯◯ LOW ^a^	lower scores indicate improvement
Aggression	–	SMD **0.29 lower** (1.08 lower to 0.49 higher)	–	25 (1 RCT)	⨁⨁◯◯ LOW ^a^	lower scores indicate improvement
Irritability	–	SMD **0.02 lower** (0.42 lower to 0.38 higher)	–	146 (5 RCTs)	⨁⨁◯◯ LOW ^a^	lower scores indicate improvement
Anxiety	–	SMD **1.01 lower** (1.86 lower to 0.17 lower)	–	25 (1 RCT)	⨁◯◯◯ VERY LOW ^a,b^	lower scores indicate improvement
Adaptive functioning	–	SMD **0.49 lower** (1.2 lower to 0.22 higher)	–	59 (2 RCTs)	⨁◯◯◯ VERY LOW ^a,c,d^	lower scores indicate worsening
Social interaction	–	SMD **0.01 lower** (0.43 lower to 0.4 higher)	–	223 (6 RCTs)	⨁◯◯◯ VERY LOW ^a,e^	lower scores indicate worsening
Restricted and repetitive interests and behaviors	–	SMD **0.01 higher** (0.36 lower to 0.39 higher)	–	223 (6 RCTs)	⨁⨁◯◯ LOW ^a^	lower scores indicate improvement
Communication	–	SMD **0.05 lower** (0.5 lower to 0.4 higher)	–	223 (6 RCTs)	⨁⨁◯◯ LOW ^a^	lower scores indicate worsening

*The risk in the intervention group (and its 95% confidence interval) is based on the assumed risk in the comparison group and the relative effect of the intervention (and its 95% CI)

^a^ Downgraded of two levels because population size < 400 and there is a wide 95%CI, which includes no effect

^b^ Downgraded of one level because the measure used was the internalizing subscale of the BASC, which only indirectly measures anxiety

^c^ Downgraded of one level because one study is at high risk for incomplete outcome data and unclear risk for blinding and selective reporting

^d^ Downgraded of one level, because in one study the “social skills, parents assessed” of the subscale “adaptive skills” of the BASC was extracted

^e^ Downgraded of one level because in two studies Social interaction was analyzed by the “social withdrawal” subscale of the ABC, which relates more to behavior and indirectly to social interaction

*CI* Confidence interval, *RR* Risk ratio, *SMD* Standardized mean difference.

GRADE Working Group grades of evidence.

High certainty: We are very confident that the true effect lies close to that of the estimate of the effect.

Moderate certainty: We are moderately confident in the effect estimate: The true effect is likely to be close to the estimate of the effect, but there is a possibility that it is substantially different.

Low certainty: Our confidence in the effect estimate is limited: The true effect may be substantially different from the estimate of the effect.

Very low certainty: We have very little confidence in the effect estimate: The true effect is likely to be substantially different from the estimate of effect.

**Table 3:** Summary of Findings (SoF) for the comparison PUFAs versus healthy diet

Summary of findings:						
Should polyunsaturated fatty acids versus healthy diet be used for the treatment of children and adolescents with autism spectrum disorder?						
Patient or population: children and adolescents with autism spectrum disorder						
Setting: outpatients						
Intervention: polyunsaturated fatty acids						
Comparison: healthy diet						
Outcomes	Anticipated absolute effects^*^ (95% CI)		Relative effect (95% CI)	№ of participants (studies)	Certainty of the evidence (GRADE)	Comments
	Risk with healthy diet	Risk with polyunsaturated fatty acids				
Quality of Sleep	–	SMD **1.11 higher** (0.21 higher to 2 higher)	–	23 (1 RCT)	⨁◯◯◯ VERY LOW ^a,b^	(lower scores indicate improvement)
Aggression	–	SMD **0** (0.83 lower to 0.82 higher)	–	23 (1 RCT)	⨁◯◯◯ VERY LOW ^a,c^	(lower scores indicate improvement)
Anxiety	–	SMD **0.16 lower** (0.99 lower to 0.66 higher)	–	23 (1 RCT)	⨁◯◯◯ VERY LOW ^a,c^	(lower scores indicate improvement)
Attention	–	SMD **0.53 lower** (1.37 lower to 0.31 higher)	–	23 (1 RCT)	⨁◯◯◯ VERY LOW ^a,c^	(lower scores indicate improvement)
Social interaction	–	SMD **0.81 lower** (1.67 lower to 0.05 higher)	–	23 (1 RCT)	⨁◯◯◯ VERY LOW ^a,c^	(lower scores indicate improvement)
Communication	–	SMD **0.36 higher** (0.47 lower to 1.19 higher)	–	23 (1 RCT)	⨁◯◯◯ VERY LOW ^a,c^	(lower scores indicate worsening)

*The risk in the intervention group (and its 95% confidence interval) is based on the assumed risk in the comparison group and the relative effect of the intervention (and its 95% CI)

^a^ Downgraded of two levels because the study was at high risk of bias for random sequence generation and performance bias and unclear risk of bias for allocation concealment, incomplete outcome data and reporting of data

^b^ Downgraded of one level because the sample size is very small and the 95%CI for SMD goes from small effect (0.21) to a very large effect (2)

^c^ Downgraded of two levels because the sample size is very small and the 95%CI includes no effect

*CI* Confidence interval, *RR* Risk ratio, *SMD* Standardized mean difference.

GRADE Working Group grades of evidence.

High certainty: We are very confident that the true effect lies close to that of the estimate of the effect.

Moderate certainty: We are moderately confident in the effect estimate: The true effect is likely to be close to the estimate of the effect, but there is a possibility that it is substantially different.

Low certainty: Our confidence in the effect estimate is limited: The true effect may be substantially different from the estimate of the effect.

Very low certainty: We have very little confidence in the effect estimate: The true effect is likely to be substantially different from the estimate of effect.

PUFAs compared to placebo did not significantly reduce aggression (SMD -0.29, 95% CI − 1.08 to 0.49; low certainty of evidence) and hyperactivity (SMD -0.27, 95% CI − 0.60 to 0.06; low certainty of evidence). PUFAs seemed superior compared to placebo in reducing anxiety in individuals with ASD (SMD -1.01, 95% CI − 1.86 to − 0.17; very low certainty of evidence). PUFAs compared to placebo not significantly worsened adaptive functioning (SMD -0.49, 95% CI − 1.20 to 0.22; very low certainty of evidence). PUFAs were similar to placebo on irritability (SMD -0.02, 95% CI − 0.42 to 0.38; low certainty of evidence), restricted and repetitive interests and behaviors (SMD 0.01, 95% CI − 0.36 to 0.39; low certainty of evidence) and communication (SMD -0.05, 95% CI − 0.50 to 0.40; low certainty of evidence). We did not find any study on the effect of PUFAs compared to placebo on other critical or important outcomes such as quality of sleep, self-harm, attention, and hyperactivity and disruptive behaviors coexistent with core symptoms.

The results for the comparison between PUFA and healthy diet were obtained from a single RCT [53]. We are uncertain whether PUFAs compared to healthy diet are efficacious on reducing anxiety (SMD -0.16, 95% CI − 0.99 to 0.66; very low certainty of evidence), aggression (SMD 0.00, 95% CI − 0.83 to 0.82; very low certainty of evidence), social interaction (SMD -0.81, 95% CI − 1.67 to 0.05; very low certainty of evidence), attention (SMD -0.53, 95% CI − 1.37 to 0.31; very low certainty of evidence), and communication (SMD 0.36, 95% CI − 0.47 to 1.19; very low certainty of evidence). We found a significant, large effect size of PUFAs when compared to healthy diet in worsening quality of sleep (SMD 1.11, 95% CI 0.21 to 2.00) with a very low certainty of evidence, while we did not find any study comparing PUFAs versus healthy diet on hyperactivity, self-harm, irritability, adaptive functioning, restricted and repetitive interests and behaviors, and hyperactivity and disruptive behaviors coexistent with core symptoms.

Regarding PUFAs safety profile, we found no difference in the Risk Ratio (RR) of experiencing an adverse event in individuals assigned to PUFAs arms compared to placebo (RR 1.54, 95% CI 0.79 to 2.97), with an estimate of 71 more events per 1000 individuals (95% CI 28 fewer to 256 more; low certainty of evidence), while we are uncertain whether PUFAs increase adverse events when compared to a healthy diet (RR 1.30, 95% CI 0.60 to 2.82; very low certainty of evidence). Discontinuation due to any cause may be similar across PUFAs and placebo arms (RR 1.06, 95% CI 0.56 to 2.03; low certainty of evidence), while no study was found reporting attrition for the comparison PUFAs versus healthy diet.

### Risk of Bias

The risk of bias assessment of the included studies is shown in the Risk of Bias Summary (Additional file 2). Three studies [47, 49, 51] were judged as low risk of bias for all the considered domains. Only one study [53] was characterized by a high risk of bias for random sequence generation and for blinding, while another study [52] presented a high risk of bias for incomplete outcome data. One study [48] presented an unclear risk of bias in four domains (random sequence generation, allocation concealment, blinding, and other sources of bias), while the remaining two studies [12, 46] presented an unclear risk of bias for incomplete outcome data. We included an insufficient number of studies to perform a meaningful presentation of publication bias through funnel plots [54].

Heterogeneity (I^2^) across considered outcomes was between 0 and 58% in the comparison of PUFAs versus placebo, while there was no heterogeneity in the comparison between PUFAs and healthy diet since only one trial was included (Additional file 3 and Additional file 4). Our judgment on inconsistency is shown in Additional file 5 and Additional file 6.

## Discussion

We conducted a systematic review and meta-analysis on efficacy and tolerability of the use of PUFAs compared to placebo or a healthy diet for children and adolescents with ASD. We found that despite some increase in number and quality of studies on PUFAs for children and adolescents with ASD over time (six RCTs were published in the last five years), results remained preliminary. PUFAs did not show evidence of effect for children and adolescents with ASD and the certainty of evidence as measured with the GRADE was low to very low.

This systematic review and meta-analysis is based on 9 studies, including 201 children and adolescents randomly assigned to PUFAs, 161 to placebo and 13 to healthy diet. Our systematic search was comprehensive and to our knowledge, this is the most up-to-date synthesis of data on this field.

This study has some limitations. First, the comparison healthy diet had a small number of studies included (only one RCT) and a very small number of participants (13 children and adolescents with ASD), limiting the evidence and the generalizability of the results.

Second, some of the outcomes which were considered as critical or important were not assessed by any study (i.e. for the comparison PUFAs versus placebo: quality of sleep, self-harm, attention, hyperactivity and disruptive behaviors coexistent with core symptoms; for PUFAs versus healthy diet: hyperactivity, self-harm, irritability, adaptive functioning, restricted and repetitive interests and behaviors, hyperactivity and disruptive behaviors coexistent with core symptoms).

Third, dosage varied greatly, from a minimum of 200 mg/day [52] to a maximum of 1540 mg/day [44], making it difficult to address causal inference. International agencies as well do not fully agree on the dietary recommended intake for PUFAs. As for omega-3 fatty acids for infants, the WHO suggests 400 mg per 10 kg body weight [15, 55], while the International Scientific Society of Fatty Acids and Lipids (ISSFAL) suggests 350–750 mg per 10 kg body weight [56]. Regarding the maximum tolerable dose of omega-3, the Food and Drug Administration (FDA) recommends not to take more than 3 g/day of EPA and DHA, of which up to 2d/day through supplements [57]. The daily limitation aims to limit the intake of fat-soluble vitamins, such as Vitamin A and Vitamin D [15, 58]. The Institute of Medicine (IOM) has not established a tolerable Upper Intake Level (UL) for omega-3 intake, but has shown that high doses (more than 900 mg/day of EPA plus 600 mg/day of DHA) may reduce the immune response, while doses between 2 and 15 g of EPA and/or DHA may have negative effects on coagulation, promoting bleeding [59]. According to the European Food Safety Authority (EFSA), however, supplementation with doses up to 5 g/day of EPA and/or DHA would be safe, as no side effects have been found regarding bleeding and immune response [60].

Fourth, our reviews did not take into account the difference between nutraceuticals and pharmacological products. Indeed, a recent systematic review highlighted differences in safety between nutraceuticals and pharmacological PUFAs, pointing out that prescribed pharmacological products are supported by robust clinical development and safety monitoring programs, while nutraceuticals are not required to demonstrate safety or efficacy before marketing [61]. Nutraceuticals may also contain potentially harmful components, including other lipids, cholesterol and toxins, and are not produced in Good Manufacturing Practice (GMP), while pharmacological products contain high purity DHA and/or EPA [61, 62].

Fifth, we did not prospectively register the protocol for our systematic review, and this is a study limitation. However, the clinical question was formulated by a multidisciplinary panel of experts, and the methodology followed for the development of the systematic review was based on the manual developed and published by the ISS [18, 63].

Previous systematic reviews on PUFAs for children and adolescents with ASD included respectively two RCTs [64], four RCTs [65], and five RCTs [66] concluding that there was no evidence of effect [64, 66] or that PUFAs could potentially improve some ASD symptoms [65]. The differences between our findings and the one of the meta-analysis of RCTs by Mazahery et al. [65], especially when considering the efficacy of PUFAs on communication outcome they found (4 RCTs; MD -1.96, 95% CI − 3.57 to − 0.34), could be partially due to the different methods used: the authors performed their analyses extracting the mean change and SD of change from baseline to endpoint. Moreover, we used the Standardized Mean Difference to pool data from assessed through different scales, while Mazahery et al. extracted only data regarding ABC subscales. Horvath et al. [66] performed different meta-analyses for each instrument used to assessed the outcomes of interest; they found PUFAs to be efficacious in improving lethargy-social withdrawal (2 RCTs; MD 1.98, 95% CI 0.32 to 3.63) when assessed with the ABC, and daily-living (1 RCT; MD 6.2, 95% CI 0.37 to 12.03) as assessed by VABS. Also, the authors found the PUFAs to worsen externalizing behavior (2 RCTs; MD -6.22, 95% CI − 10.9 to − 1.59) and social skills (1 RCT; MD -7.0, 95% CI − 13.62 to − 0.38) as assessed through BASC. The most recent RCT published [46], not included in the previous systematic reviews, suggested that PUFAs could improve some core symptoms of ASD, but its findings, when pooled with other RCTs results, did not translate into statistical significance for any outcome in our meta-analysis. These mixed findings are in line with the very low and low certainty of evidence found in our study by using the GRADE. Notwithstanding the publication of numerous RCTs in recent years comparing PUFAs against placebo, their sample size was always small. The consequence was that the most frequent reason for lowering the certainty of evidence in our systematic review was the insufficient sample size. It therefore appears necessary to conduct larger RCTs to establish the efficacy of PUFA in this population; this would be even more true if the size of the effect to be highlighted were small.

Although the efficacy of PUFAs in children and adolescents with ASD is still controversial, PUFAs are administered to a portion of this population ranging from 18 to 51% [67, 68], probably thanks to the fact that costs and difficulties of implementation appear negligible [69], and safety concerns seem small [65]. Also the rationale behind the administration of PUFAs in individuals with ASD, i.e. that the observation of reduced plasma concentrations of EPA and DHA in meta-analyses of case-control studies would be due to inefficient or disrupted metabolism, could instead be due to the action of confounding factors, i.e. selective diets [65], and needs to be demonstrated through good quality studies, controlled for possible confounders.

## Conclusions

In conclusion, we found no evidence of efficacy for PUFAs versus placebo on hyperactivity, aggression, irritability, adaptive functioning, social interaction, restricted and repetitive interests and behaviours, communication, with very low to low certainty of evidence, evidence of efficacy on anxiety only, with very low certainty of evidence and evidence of a negative effect on quality of sleep, with very low certainty of evidence. We found no evidence of efficacy of PUFAs versus healthy diet on aggression, anxiety, attention, social interaction, communication with very low certainty of evidence and we found efficacy on quality of sleep, with very low certainty of evidence. No clinical recommendation can be suggested at the present time. We do not believe the evidence is strong enough to allow the construction of a phase III trial, while phase II, dose-findings trials are necessary to ascertain the dose and the effect of PUFAs for children and adolescents with ASD.

## Supplementary information

**Additional file 1.** Search strategy and results

**Additional file 2.** Risk of Bias Summary

**Additional file 3.** Forest plots of comparisons between PUFAs and Placebo

**Additional file 4.** Forest plots of comparisons between PUFAs and healthy diet

**Additional file 5.** Evidence profile - PUFAs versus Placebo

**Additional file 6.** Evidence profile - PUFAs versus healthy diet

**Additional file 7.** References for included and excluded trials

**Additional file 8.** PRISMA checklist

## Data Availability

All data supporting our findings is contained within the manuscript and the additional files. The authors and can be contacted at f.decrescenzo@deplazio.it. (FDC) for further clarification, if required.

## Abbreviations

ABC: Aberrant Behavior Checklist
ADHD: attention deficit hyperactivity disorder
ASD: autism spectrum disorder
BASC: Behavior Assessment System for Children
CGI-I: Clinical Global Impression-Improvement scale
CGI-S: Clinical Global Impression-Severity scale
CI: Confidence interval
DHA: Docosahexaenoic acid
EPA: Eicosapentaenoic acid
EVT: Expressive Vocabulary Test
GRADE: Grading of Recommendations Assessment, Development and Evaluation
MSEL: Mullen Scales of Early Learning
PLS: Preschool Language Scale
PPVT: Peabody Picture Vocabulary Test
PUFA: polyunsaturated fatty acid
RCT: randomized controlled trial
RR: risk ratio
SMD: standardized mean difference
SRS: Social Responsiveness Scale
VABS: Vineland Adaptive Behavior Scale
