# Supplementary material for: Impact of polyunsaturated fatty acids on patient-important outcomes in children and adolescents with autism spectrum disorder: a systematic review
Source: Health Qual Life Outcomes. 2020 Feb 17;18:28. doi: 10.1186/s12955-020-01284-5 (PMC7026962; doi:10.1186/s12955-020-01284-5)
Supplement: Supplementary file 2 — Additional file 2. Risk of Bias Summary [file 12955_2020_1284_MOESM2_ESM.pptx]

## Slide 1
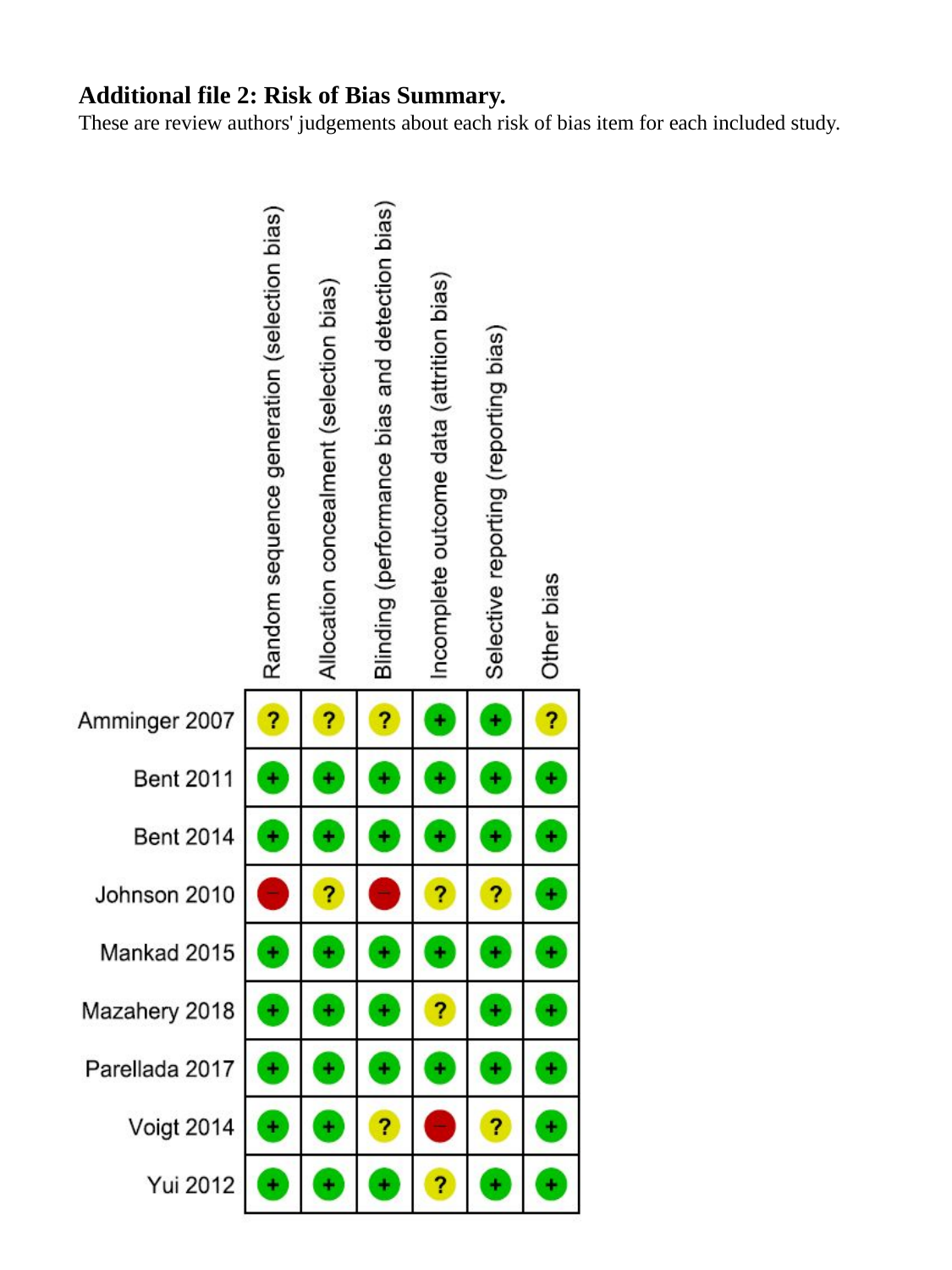

Additional file 2: Risk of Bias Summary.
These are review authors' judgements about each risk of bias item for each included study.
