# Supplementary material for: Impact of polyunsaturated fatty acids on patient-important outcomes in children and adolescents with autism spectrum disorder: a systematic review
Source: Health Qual Life Outcomes. 2020 Feb 17;18:28. doi: 10.1186/s12955-020-01284-5 (PMC7026962; doi:10.1186/s12955-020-01284-5)

Additional file 3: Forest plots of comparisons between PUFAs and Placebo

# Adaptive Functioning.

Forest plot of comparison between PUFAs and Placebo.


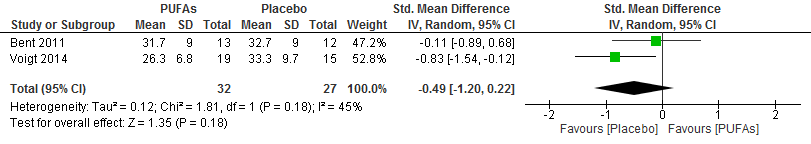


# Aggression.

Forest plot of comparison between PUFAs and Placebo.


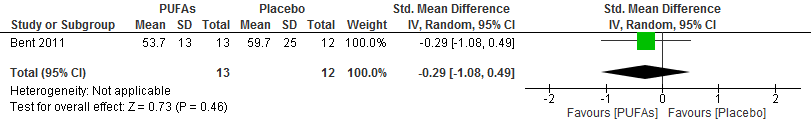


# Anxiety.

Forest plot of comparison between PUFAs and Placebo.


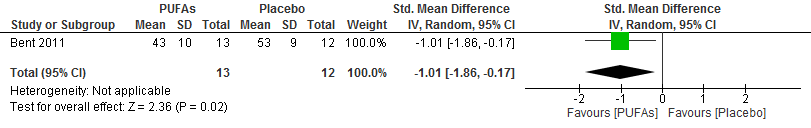


# Communication.

Forest plot of comparison between PUFAs and Placebo.


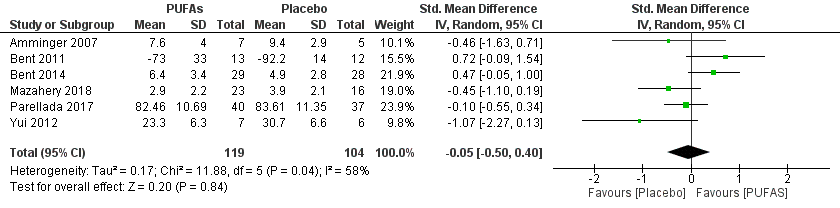


# Discontinuation due to any cause.

Forest plot of comparison between PUFAs and Placebo.


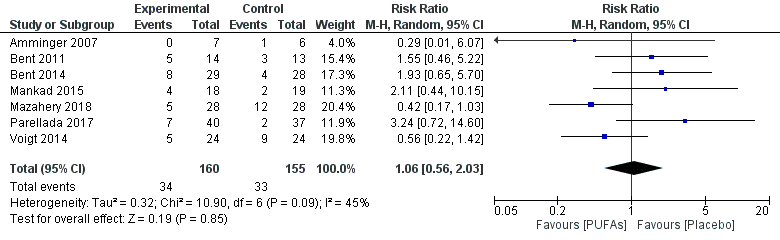


Hyperactivity.

Forest plot of comparison between PUFAs and Placebo.

^
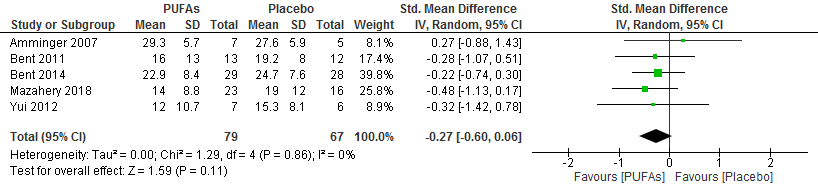
^

# Irritability.

Forest plot of comparison between PUFAs and Placebo.

^
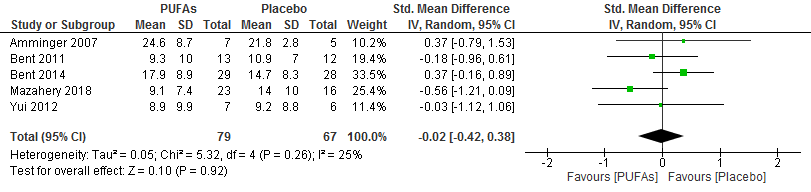
^

Number of adverse events.

Forest plot of comparison between PUFAs and Placebo.


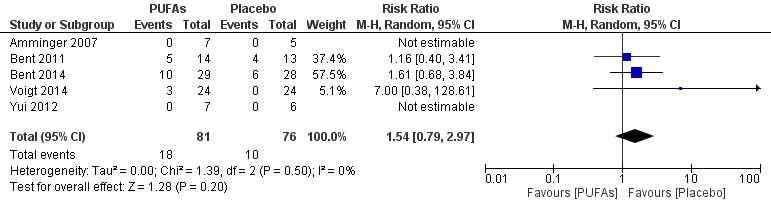


Restricted and repetitive interests and behaviors.

Forest plot of comparison between PUFAs and Placebo.


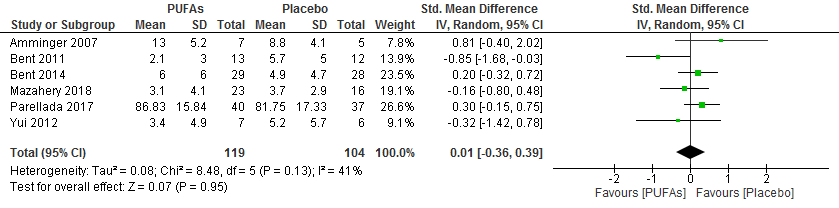


# Social Interaction.

Forest plot of comparison between PUFAs and Placebo.


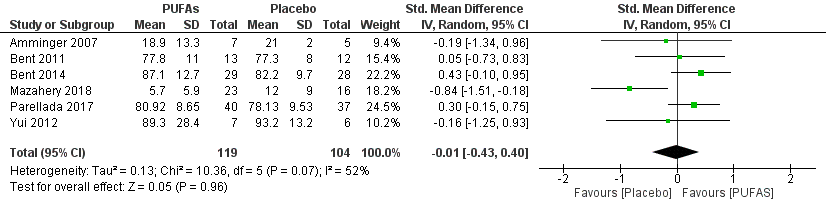

Supplement: Supplementary file 3 — Additional file 3. Forest plots of comparisons between PUFAs and Placebo [file 12955_2020_1284_MOESM3_ESM.docx]
