# Supplementary material for: Impact of polyunsaturated fatty acids on patient-important outcomes in children and adolescents with autism spectrum disorder: a systematic review
Source: Health Qual Life Outcomes. 2020 Feb 17;18:28. doi: 10.1186/s12955-020-01284-5 (PMC7026962; doi:10.1186/s12955-020-01284-5)

Additional file 4: Forest plots of comparisons between PUFAs and placebo.

# Aggression.


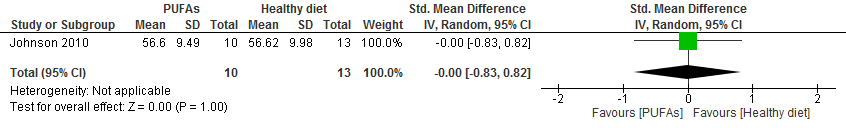


# Anxiety.

Forest plot of comparison between PUFAs and Healthy diet .


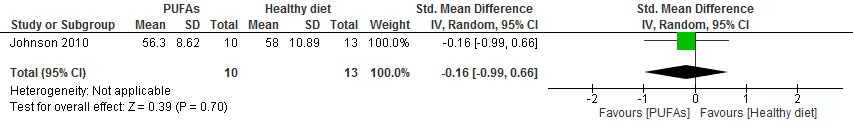


# Attention.

Forest plot of comparison between PUFAs and Healthy diet.


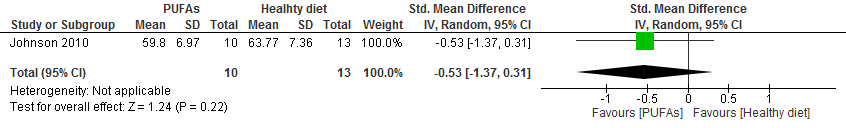


# Communication.

Forest plot of comparison between PUFAs and Healthy diet.


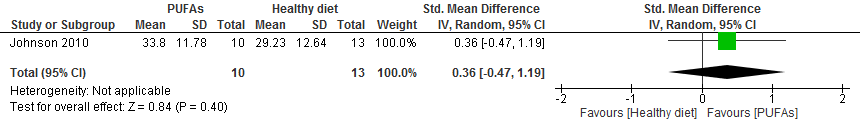


# Number of adverse events.

Forest plot of comparison between PUFAs and Healthy diet.


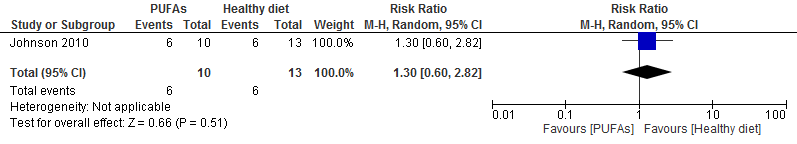


# Sleep quality.

Forest plot of comparison between PUFAs and Healthy diet.


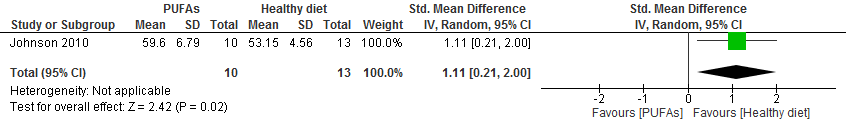


# Social Interaction.

Forest plot of comparison between PUFAs and Healthy diet.


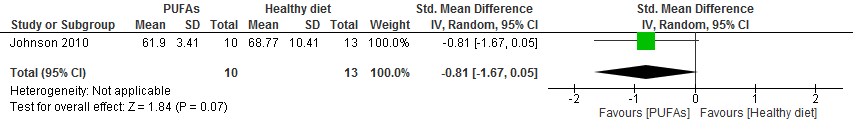

Supplement: Supplementary file 4 — Additional file 4. Forest plots of comparisons between PUFAs and healthy diet [file 12955_2020_1284_MOESM4_ESM.docx]
