# Supplementary material for: Impact of polyunsaturated fatty acids on patient-important outcomes in children and adolescents with autism spectrum disorder: a systematic review
Source: Health Qual Life Outcomes. 2020 Feb 17;18:28. doi: 10.1186/s12955-020-01284-5 (PMC7026962; doi:10.1186/s12955-020-01284-5)
Supplement: Supplementary file 5 — Additional file 5. Evidence profile - PUFAs versus Placebo [file 12955_2020_1284_MOESM5_ESM.docx]

**Additional file 5**

**Evidence profile – PUFAs versus placebo**

**Author(s)**:

**Date**:

**Question**: Should polyunsaturated fatty acids versus placebo be used for the treatment of children and adolescents with autism spectrum disorder?

**Setting**:

**Bibliography**:

| **Certainty assessment** | | | | | | | **№ of patients** | | **Effect** | | **Certainty** | **Importance** |
| --- | --- | --- | --- | --- | --- | --- | --- | --- | --- | --- | --- | --- |
| **№ of studies** | **Study design** | **Risk of bias** | **Inconsistency** | **Indirectness** | **Imprecision** | **Other considerations** | **Polyunsaturated fatty acids** | **Placebo** | **Relative (95% CI)** | **Absolute (95% CI)** |  |  |
| **Discontinuation due to any cause** | | | | | | | | | | | | |
| 7 | randomised trials | not serious | not serious | not serious | very serious ^a^ | none | 34/160 (21.3%) | 33/155 (21.3%) | **RR 1.06** (0.56 to 2.03) | **13 more per 1.000** (from 94 fewer to 219 more) | ⨁⨁◯◯ LOW | NOT IMPORTANT |
| **Hyperactivity (lower scores indicate improvement)** | | | | | | | | | | | | |
| 5 | randomised trials | not serious | not serious | not serious | very serious ^b^ | none | 79 | 67 | - | SMD **0.27 lower** (0.6 lower to 0.06 higher) | ⨁⨁◯◯ LOW | CRITICAL |
| **Sleep quality** | | | | | | | | | | | | |
| 0 |  |  |  |  |  |  | 0 | 0 | - | see comment | - | CRITICAL |
| **Self-harm** | | | | | | | | | | | | |
| 0 |  |  |  |  |  |  | 0 | 0 | - | see comment | - | CRITICAL |
| **Aggression (lower scores indicate improvement)** | | | | | | | | | | | | |
| 1 | randomised trials | not serious | not serious | not serious | very serious ^b^ | none | 13 | 12 | - | SMD **0.29 lower** (1.08 lower to 0.49 higher) | ⨁⨁◯◯ LOW | CRITICAL |
| **Irritability (lower scores indicate improvement)** | | | | | | | | | | | | |
| 5 | randomised trials | not serious | not serious | not serious | very serious ^b^ | none | 79 | 67 | - | SMD **0.02 lower** (0.42 lower to 0.38 higher) | ⨁⨁◯◯ LOW | CRITICAL |
| **Anxiety (lower scores indicate improvement)** | | | | | | | | | | | | |
| 1 | randomised trials | not serious | not serious | serious ^c^ | very serious ^b^ | none | 13 | 12 | - | SMD **1.01 lower** (1.86 lower to 0.17 lower) | ⨁◯◯◯ VERY LOW | CRITICAL |
| **Attention** | | | | | | | | | | | | |
| 0 |  |  |  |  |  |  | 0 | 0 | - | see comment | - | CRITICAL |
| **Adaptive functioning (lower scores indicate worsening)** | | | | | | | | | | | | |
| 2 | randomised trials | serious ^d^ | not serious | serious ^e^ | very serious ^b^ | none | 32 | 27 | - | SMD **0.49 lower** (1.2 lower to 0.22 higher) | ⨁◯◯◯ VERY LOW | CRITICAL |
| **Social interaction (lower scores indicate worsening)** | | | | | | | | | | | | |
| 4 | randomised trials | not serious | not serious | serious ^f^ | very serious ^b^ | none | 89 | 83 | - | SMD **0.27 higher** (0.03 lower to 0.57 higher) | ⨁◯◯◯ VERY LOW | IMPORTANT |
| **Restricted and repetitive interests and behaviors (lower scores indicate improvement)** | | | | | | | | | | | | |
| 6 | randomised trials | not serious | not serious | not serious | very serious ^b^ | none | 119 | 104 | - | SMD **0.01 higher** (0.36 lower to 0.39 higher) | ⨁⨁◯◯ LOW | IMPORTANT |
| **Communication (lower scores indicate worsening)** | | | | | | | | | | | | |
| 6 | randomised trials | not serious | not serious | not serious | very serious ^b^ | none | 119 | 104 | - | SMD **0.05 SD lower** (0.5 lower to 0.4 higher) | ⨁⨁◯◯ LOW | IMPORTANT |
| **Hyperactivity and disruptive behaviors coexistent with core symptoms** | | | | | | | | | | | | |
| 0 |  |  |  |  |  |  | 0 | 0 | - | see comment | - | IMPORTANT |
| **Number of adverse events** | | | | | | | | | | | | |
| 5 | randomised trials | not serious | not serious | not serious | very serious ^a^ | none | 18/81 (22.2%) | 10/76 (13.2%) | **RR 1.54** (0.79 to 2.97) | **71 more per 1.000** (from 28 fewer to 259 more) | ⨁⨁◯◯ LOW | NOT IMPORTANT |

**CI:** Confidence interval; **RR:** Risk ratio; **SMD:** Standardised mean difference

#### Explanations

a. Downgraded of two levels because optimal information size (OIS) not met and there is a wide 95%CI, which includes no effect

b. Downgraded of two levels because population size <400 and there is a wide 95%CI, which includes no effect

c. Downgraded of one level because the measure used was the internalizing subscale of the BASC, which only indirectly measures anxiety

d. Downgraded of one level because one study is at high risk for incomplete outcome data and unclear risk for blinding and selective reporting

e. Downgraded of one level, because in one study the "social skills, parents assessed" of the subscale "adaptive skills" of the BASC was extracted

f. Downgraded of one level because in two studies Social interaction was analysed by the "inappropriate speech" subscale of the ABC, which relates more to behaviour and indirectly to social interaction
