# Supplementary material for: Impact of polyunsaturated fatty acids on patient-important outcomes in children and adolescents with autism spectrum disorder: a systematic review
Source: Health Qual Life Outcomes. 2020 Feb 17;18:28. doi: 10.1186/s12955-020-01284-5 (PMC7026962; doi:10.1186/s12955-020-01284-5)
Supplement: Supplementary file 6 — Additional file 6. Evidence profile - PUFAs versus healthy diet [file 12955_2020_1284_MOESM6_ESM.docx]

**Additional file 6**

**Evidence profile – PUFAs versus healthy diet**

**Author(s)**:

**Date**:

**Question**: Should polyunsaturated fatty acids versus healthy diet be used for the treatment of children and adolescents with autism spectrum disorder?

**Setting**:

**Bibliography**:

| **Certainty assessment** | | | | | | | **№ of patients** | | **Effect** | | **Certainty** | **Importance** |
| --- | --- | --- | --- | --- | --- | --- | --- | --- | --- | --- | --- | --- |
| **№ of studies** | **Study design** | **Risk of bias** | **Inconsistency** | **Indirectness** | **Imprecision** | **Other considerations** | **Polyunsaturated fatty acids** | **Healthy diet** | **Relative (95% CI)** | **Absolute (95% CI)** |  |  |
| **Discontinuation due to any cause** | | | | | | | | | | | | |
| 0 |  |  |  |  |  |  | 0/0 | 0/0 | not pooled | see comment | - | NOT IMPORTANT |
| **Hyperactivity** | | | | | | | | | | | | |
| 0 |  |  |  |  |  |  | 0 | 0 | - | see comment | - | CRITICAL |
| **Sleep quality (lower scores indicate improvement)** | | | | | | | | | | | | |
| 1 | randomised trials | very serious ^a^ | not serious | not serious | serious ^b^ | none | 10 | 13 | - | SMD **1.11 higher** (0.21 higher to 2 higher) | ⨁◯◯◯ VERY LOW | CRITICAL |
| **Self-harm** | | | | | | | | | | | | |
| 0 |  |  |  |  |  |  | 0 | 0 | - | see comment | - | CRITICAL |
| **Aggression (lower scores indicate improvement)** | | | | | | | | | | | | |
| 1 | randomised trials | very serious ^a^ | not serious | not serious | very serious ^c^ | none | 10 | 13 | - | SMD **0**  (0.83 lower to 0.82 higher) | ⨁◯◯◯ VERY LOW | CRITICAL |
| **Irritability** | | | | | | | | | | | | |
| 0 |  |  |  |  |  |  | 0 | 0 | - | see comment | - | CRITICAL |
| **Anxiety (lower scores indicate improvement)** | | | | | | | | | | | | |
| 1 | randomised trials | very serious ^a^ | not serious | not serious | very serious ^c^ | none | 10 | 13 | - | SMD **0.16 lower** (0.99 lower to 0.66 higher) | ⨁◯◯◯ VERY LOW | CRITICAL |
| **Attention (lower scores indicate improvement)** | | | | | | | | | | | | |
| 1 | randomised trials | very serious ^a^ | not serious | not serious | very serious ^c^ | none | 10 | 13 | - | SMD **0.53 lower** (1.37 lower to 0.31 higher) | ⨁◯◯◯ VERY LOW | CRITICAL |
| **Adaptive functioning** | | | | | | | | | | | | |
| 0 |  |  |  |  |  |  | 0 | 0 | - | see comment | - | CRITICAL |
| **Social interaction (lower scores indicate improvement)** | | | | | | | | | | | | |
| 1 | randomised trials | very serious ^a^ | not serious | not serious | very serious ^c^ | none | 10 | 13 | - | SMD **0.81 lower** (1.67 lower to 0.05 higher) | ⨁◯◯◯ VERY LOW | IMPORTANT |
| **Restricted and repetitive interests and behaviors** | | | | | | | | | | | | |
| 0 |  |  |  |  |  |  | 0 | 0 | - | see comment | - | IMPORTANT |
| **Communication (lower scores indicate worsening)** | | | | | | | | | | | | |
| 1 | randomised trials | very serious ^a^ | not serious | not serious | very serious ^c^ | none | 10 | 13 | - | SMD **0.36 higher** (0.47 lower to 1.19 higher) | ⨁◯◯◯ VERY LOW | IMPORTANT |
| **Hyperactivity and disruptive behaviors coexistent with core symptoms** | | | | | | | | | | | | |
| 0 |  |  |  |  |  |  | 0 | 0 | - | see comment | - | IMPORTANT |
| **Number of adverse events** | | | | | | | | | | | | |
| 1 | randomised trials | very serious ^a^ | not serious | not serious | very serious ^c^ | none | 6/10 (60.0%) | 6/13 (46.2%) | **RR 1.30** (0.60 to 2.82) | **138 more per 1.000** (from 185 fewer to 840 more) | ⨁◯◯◯ VERY LOW | NOT IMPORTANT |

**CI:** Confidence interval; **RR:** Risk ratio; **SMD:** Standardised mean difference

#### Explanations

a. Downgraded of two levels because the study was at high risk of bias for random sequence generation and performance bias and unclear risk of bias for allocation concealment, incomplete outcome data and reporting of data

b. Downgraded of one level because the sample size is very small and the 95%CI for SMD goes from small effect (0.21) to a very large effect (2)

c. Downgraded of two levels because the sample size is very small and the 95%CI includes no effect
