# Supplementary material for: Impact of polyunsaturated fatty acids on patient-important outcomes in children and adolescents with autism spectrum disorder: a systematic review
Source: Health Qual Life Outcomes. 2020 Feb 17;18:28. doi: 10.1186/s12955-020-01284-5 (PMC7026962; doi:10.1186/s12955-020-01284-5)
Supplement: Supplementary file 7 — Additional file 7. References for included and excluded trials [file 12955_2020_1284_MOESM7_ESM.docx]

# Additional file 7

**References for included and excluded trials**

# References for included trials

- Amminger 2007
  - Amminger GP, Berger GE, Schafer MR, Klier C, Friedrich MH, Feucht M. Omega-3 fatty acids supplementation in children with autism: A double-blind randomized, placebo-controlled pilot study. Biological Psychiatry 2007;61:551-3.
- Bent 2011
  - * Bent S, Bertoglio K, Ashwood P, Bostrom A, Hendren RL. A pilot randomized controlled trial of omega-3 fatty acids for autism spectrum disorder. Journal of Autism and Developmental Disorders 2011;41(5):545-54
  - NCT00786799. Omega-3 Fatty Acids for Autism Treatment. clinicaltrial.gov (first posted 6^th^ November 2008).
- Bent 2014
  - * Bent S, Hendren RL, Zandi T, Law K, Choi JE, Widjaja F, Kalb L, Nestle J, Law P. Internet-based, randomized, controlled trial of omega-3 fatty acids for hyperactivity in autism. J Am Acad Child Adolesc Psychiatry 2014;53(6):658-66.
  - NCT01694667. Omega-3 Fatty Acids for Hyperactivity Treatment in Autism Spectrum Disorder. clinicaltrial.gov (first posted 27^th^ September 2012).
- Johnson 2010
  - Johnson CR, Handen BL, Zimmer M, Sacco K. Polyunsaturated fatty acid supplementation in young children with autism. Journal of Developmental and Physical Disabilities 2010;22:1-10.
- Mankad 2015
  - * Mankad D, Dupuis A, Smile S, Roberts W, Brian J, Lui T, Genore L, Zaghloul D, Iaboni A, Marcon PM, Anagnostou E. A randomized, placebo controlled trial ofomega-3 fatty acids in the treatment of young children with autism. Mol Autism 2015;6:18.
  - NCT01248728. Omega-3 Fatty Acids For Treatment Of Young Children With Autism (OMG). clinicaltrial.gov (first posted 18^th^ May 2016).
- Mazahery 2018
  - * Mazahery H, Conlon CA, Beck KL, Mugridge O, Kruger MC, Stonehouse W, Camargo CA, Meyer BJ, Jones B, von Hurst PR. A randomised controlled trial of vitamin D and omega-3 long chain polyunsaturated fatty acids in the treatment of irritability and hyperactivity among children with Autism Spectrum Disorder. Journal of Steroid Biochemistry and Molecular Biology 2018. DOI: <https://doi.org/10.1016/j.jsbmb.2018.10.017>
  - Mazahery H, Conlon C, Beck KL, Kruger MC, Stonehouse W, Camargo CA Jr, Meyer BJ, Tsang B, Mugridge O, von Hurst PR. Vitamin D and omega-3 fatty acid supplements in children with autism spectrum disorder: a study protocol for a factorial randomised, double-blind, placebo-controlled trial. Trials 2016;17(1):295.
  - ACTRN12615000144516. Effect of Vitamin D and Omega-3 Fatty Acid Supplements on behavioural measures in Children with Autism Spectrum Disorder (ASD): A randomised, double-blind, placebo-controlled trial. anzctr.org.au/Trial (first registered 16^th^ February 2015).
- Parellada 2017
  - Moreno C, Calvo-Escalona R, Gutierrez S, Graell M, Romo J, Dorado ML, Giraldez ML, Llorente C, Arango C, Parellada M. Effect of omega-3 polyunsaturated fatty acids on oxidative stress in children and adolescents with autism spectrum disorders. European neuropsychopharmacology 2014;24:S725.
  - * Parellada M, Llorente C, Calvo R, Gutierrez S, Lázaro L, Graell M, GuisasolaM, Dorado ML, Boada L, Romo J, Dulin E, Sanz I, Arango C, Moreno C. Randomized trial of omega-3 for autism spectrum disorders: Effect on cell membrane composition and behavior. Eur Neuropsychopharmacol 2017;27(12):1319-30.
- Voigt 2014
  - * Voigt RG, Mellon MW, Katusic SK, Weaver AL, Matern D, Mellon B, Jensen CL,Barbaresi WJ. Dietary docosahexaenoic acid supplementation in children with autism. Journal of Pediatric Gastroenterology and Nutrition 2014;58(6):715-22.
  - NCT00577447. Docosahexaenoic Acid in the Treatment of Autism. clinicaltrial.gov (first posted 20^th^ December 2007).
- Yui 2012
  - * Yui K, Koshiba M, Nakamura S, Kobayashi Y. Effects of large doses of arachidonic acid added to docosahexaenoic acid on social impairment in individuals with autism spectrum disorders: a double-blind, placebo-controlled, randomized trial. Journal of clinical psychopharmacology 2012;32(2):200-6.
  - Yui K, Koshiba K, Nakamura S. Effects of adding large doses of arachidonic acid to docosahexaenoic acid on social impairment in individuals with autism spectrum disorders. Current psychopharmacology 2013;2(1):84-90.
  - NCT01154894. Dietary Fatty Acids Improve Social Impairment in Autism Spectrum Disorders (Fatty acid). clinicaltrial.gov (first posted 1^st^ July 2010).

# References for excluded studies

- Adams 2018
  - Adams JB, Audhya T, Geis E, Gehn E, Fimbres V, Pollard EL, Mitchell J, Ingram J, Hellmers R, Laake D, Matthews JS, Li K, Naviaux JC, Naviaux RK, Adams RL,Coleman DM, Quig DW. Comprehensive Nutritional and Dietary Intervention forAutism Spectrum Disorder-A Randomized, Controlled 12-Month Trial. Nutrients 2018;10(3):E369.
  - NCT02059577. Nutritional and Dietary Treatment Study for Children/Adults With Autism. clinicaltrial.gov (first posted 11^th^ Febraury 2014).
- Boone 2017
  - Boone KM, Gracious B, Klebanoff MA, Rogers LK, Rausch J, Coury DL, Keim SA. Corrigendum to "Omega-3 and -6 fatty acid supplementation and sensory processing in toddlers with ASD symptomology born preterm: a randomized controlled trial". Early Human Development 2018.
  - Boone KM, Gracious B, Klebanoff MA, Rogers LK, Rausch J, Coury DL, Keim SA.. Omega-3 and -6 fatty acid supplementation and sensory processing in toddlers with ASD symptomology born preterm: a randomized controlled trial. Early Human Development 2017;115:64-70.
  - Keim SA, Gracious B, Boone KM, Klebanoff MA, Rogers LK, Rausch J, Coury DL,Sheppard KW, Husk J, Rhoda DA.. ω-3 and ω-6 Fatty Acid Supplementation May Reduce Autism Symptoms Based on Parent Report in Preterm Toddlers.. J Nutr 2018;148(2):227-35.
  - Sheppard KW, Boone KM, Gracious B, Klebanoff MA, Rogers LK, Rausch J, BartlettC, Coury DL, Keim SA.. Effect of Omega-3 and -6 Supplementation on Language in Preterm Toddlers Exhibiting Autism Spectrum Disorder Symptoms.. J Autism Dev Disord 2017;47(11):3358-69.
  - NCT01683565. Preemie Tots: A Pilot Study to Understand the Effects of Prematurity in Toddlerhood. clinicaltrial.gov (first posted: 12^th^ September 2012).
- Johnson 2003
  - Johnson SM, Hollander E. Evidence that eicosapentaenoic acid is effective in treating autism. Journal of Clinical Psychiatry 2003;64:848-9.
- Meguid 2008
  - Meguid NA, Atta HM, Gouda AS, Khalil RO. Role of polyunsaturated fatty acids in the management of Egyptian children with autism. Clinical Biochemistry 2008;41:1044-8.
- Meiri 2009
  - Meiri G, Bichovsky Y, Belmaker RH. Omega 3 fatty acid treatment in autism. Journal of Child and Adolescent Psychopharmacology 2009;19(4):449-51.
- NCT01695200
  - NCT01695200. Omega-3 Fatty Acids in Autism Spectrum Disorders. clinicaltrial.gov (first posted 27^th^ September 2012).
- NCT03757585
  - NCT03757585. Management of Emotional Dysregulation in Youth With Non-verbal Learning Disability (NVLD) and/or Autism Spectrum Disorders (ASD) Using Telepsychiatry of Complementary and Alternative Treatments. clinicaltrial.gov (first posted 29^th^ November 2018).
- Ooi 2015
  - Ooi YP, Weng SJ, Jang LY, Low L, Seah J, Teo S, Ang RP, Lim CG, Liew A, Fung DS, Sung M. Omega-3 fatty acids in the management of autism spectrum disorders: findings from an open-label pilot study in Singapore. Eur J Clin Nutr. 2015 Aug;69(8):969-71. doi: 10.1038/ejcn.2015.28. Epub 2015 Mar 25. PubMed PMID: 25804268.
- Patrick 2005
  - Patrick L, Salik R. The effect of essential fatty acid supplementation on language development and learning skills in autism and Asperger's syndrome. Autism Asperger's Digest 2005;Jan-Feb:36-7.
- Politi 2008
  - Politi P, Cena H, Comelli M, Marrone G, Allegri C, Emanuele E, et al. Behavioral effects of omega-3 fatty acid supplementation in young adults with severe autism: an open label study. Archives of Medical Research 2008;39(7):682-5.

# Ongoing studies

- NCT03550209. Fatty Acid Supplementation in Children With ASD (Omega Heroes). clinicaltrial.gov (first posted 8^th^ June 8 2018).

# Studies awaiting classification

- NCT01260961. Developing treatment, treatment validation and treatment scope in the setting of an autism (first posted 15^th^ December 2010).
- NCT01248130. Omega-3 fatty acids monotherapy in children and adolescents with autism spectrum disorders (first posted 25^th^ November 2010).
- NCT00467818. Omega 3 Fatty Acids in the Treatment of Children With Autism Spectrum Disorders (first posted 1^st^ May 2007)
- NCT03620097. Evaluate the Efficacy and Safety of DHA in the Adjuvant Treatment of Children With ASD (first posted 8^th^ August 2018).
